# *Vibrio parahaemolyticus* in a Changing World: A One Health Perspective on Epidemiology, Antimicrobial Resistance, Virulence, and Climate-Driven Risks

**DOI:** 10.3390/pathogens15101010

**Published:** 2026-09-24

**Authors:** Ivan Camilo Sanchez-Rojas, D. Katterine Bonilla-Aldana, Catherin Lorena Solarte-Jimenez, Jorge Luis Bonilla-Aldana, Lysien I. Zambrano, Acacia Alcivar-Warren, Alfonso J. Rodriguez-Morales

**Affiliations:** 1 Grupo de Investigación en Genética, Biodiversidad y Manejo de Ecosistemas (GEBIOME), Universidad de Caldas, Manizales 170004, Caldas, Colombia; ivan.sanchez@itp.edu.co; 2 College of Medicine, Korea University, Seoul 02841, Republic of Korea; dbonilla@korea.ac.kr; 3 Grupo de Investigación en Producción y Salud Animal (GIPSA), Universidad de La Amazonía, Florencia 180001, Caquetá, Colombia; catherin9420@gmail.com; 4 Grupo de Virologia, Universidad El Bosque, Bogotá DC 110121, Colombia; jlbonilla@unbosque.edu.co; 5 Instituto de Investigaciones One Health, Universidad Tecnológica Centroamericana (UNITEC), Tegucigalpa 11101, Honduras; lysienzambrano@unitec.edu; 6 Fundación para la Conservación de la Biodiversidad Acuática y Terrestre de Ecuador (FUCOBI), Quito EC1701, Ecuador; acacia.warren.rcs@gmail.com or environmentalgenomics.warren@gmail.com; 7 Environmental Genomics Inc., ONE HEALTH Epigenomics Educational Initiative, P.O. Box 196, Southborough, MA 01772, USA; 8 Faculty of Health Sciences, Universidad Científica del Sur, Lima 15046, Peru; 9 Grupo de Investigación Biomedicina, Faculty of Medicine, Fundación Universitaria Autónoma de las Américas-Institución Universitaria Visión de las Américas, Pereira 600003, Risaralda, Colombia

**Keywords:** *Vibrio parahaemolyticus*, One Health, seafood-borne infections, climate change, antimicrobial resistance, shrimp and oyster diseases, global epidemiology

## Abstract

*Vibrio parahaemolyticus* is a halophilic, Gram-negative bacterium widely distributed in marine and estuarine environments and recognized as a leading cause of seafood-associated gastroenteritis worldwide. In recent decades, its global emergence has been driven by environmental changes, particularly ocean warming, as well as intensified aquaculture and the globalization of seafood trade. Representative epidemiological data illustrate this burden: *V. parahaemolyticus* was detected in 3.11% of 79,075 diarrheal samples across 11 Chinese provinces and in 8.1% of 6951 acute-diarrhea samples in eastern China, while Zhejiang Province documented 383 outbreaks involving 4382 cases between 2010 and 2022. This narrative review provides a comprehensive overview of the epidemiology, virulence factors, antimicrobial resistance, and One Health dimensions of *V. parahaemolyticus*. The pathogen’s ability to cause disease in humans largely depends on key virulence determinants, including thermostable direct hemolysin (TDH), TDH-related hemolysin (TRH), and secretion systems that facilitate host colonization and cytotoxicity. From a One Health perspective, *V. parahaemolyticus* represents a critical interface among environmental, animal, and human health, and also causes severe diseases in aquaculture, including acute hepatopancreatic necrosis disease (AHPND) and translucent postlarval disease (TPD) in shrimp, as well as vibriosis in bivalve mollusks such as oysters, where infection may result in tissue damage and substantial larval and juvenile mortality, with important economic consequences. The increasing emergence of antimicrobial-resistant strains further complicates clinical management and highlights the role of environmental reservoirs and aquaculture practices in disseminating resistance. Additionally, climate-driven changes in temperature, salinity, and marine ecosystems are expanding this pathogen’s geographic distribution and altering its seasonal dynamics. Strengthening integrated surveillance systems, improving food safety practices, and promoting interdisciplinary research, including studies of the molecular mechanisms underlying interactions between the epigenome and *V. parahaemolyticus*, are essential to mitigate the growing risks associated with *V. parahaemolyticus*. A comprehensive One Health approach is critical to address its evolving impact on global public health and food security.

## 1. Introduction

In the current context, human health faces crucial challenges associated with climate change and the deterioration of ecosystems that threaten water integrity and food security [1]. This growing concern has been extended in various discussions by international organizations that relate the scourge of waterborne diseases and aquatic foods to Sustainable Development Goals (SDGs) 3, 6, 11, 13, and 17 [2,3]. Likewise, the World Health Organization (WHO) highlights that contaminated water contributes to the transmission of diseases such as cholera, dysentery, hepatitis A, typhoid fever, and poliomyelitis, representing a significant risk to public health and human health [4].

Cholera, caused by the bacterium *Vibrio cholerae* O1/O139, is considered the most significant waterborne disease worldwide due to the high number of human infections and deaths reported throughout the seven pandemics documented from 1817 to the present [5]. However, at least twelve non-choleric *Vibrio* species have been identified, among which *V. parahaemolyticus*, *V. mimicus*, *V. vulnificus*, *V. cholerae* non-O1/non-O139, *V. fluvialis,* and *V. alginolyticus* stand out for their clinical relevance, as they are the main cause of vibriosis in humans [6].

*Vibrio parahaemolyticus* is a heterotrophic and halophilic Gram-negative bacterium that naturally inhabits estuarine and marine environments around the world; it grows, preferably, in warm waters with temperatures equal to or greater than 15 °C and an average salinity of 2.5–3% NaCl [7]. The bacterium *V. parahaemolyticus* is responsible for one of the most important foodborne diseases worldwide, acute gastroenteritis, with shrimp, oysters, crabs, mussels, fish, and edible seaweed being the main sources of infection [8]. This pathogen is commonly associated with environments in which aquaculture activities are carried out; for this reason, it represents a significant threat to both aquatic life and human health [9]. Similarly, the importance of *V. parahaemolyticus* lies in the fact that, together with *V. cholerae* O1, it is one of the few marine bacteria with the proven ability to cause global epidemics through its spread between continents [6,10].

In 1950, from food poisoning cases in Japan, the doctor Fujino Tsunesaburo discovered the bacterium *V. parahaemolyticus* [11]. Since then, isolates in aquatic organisms, as well as several outbreaks of food poisoning and sporadic cases of infection with this bacterium, have been reported in Africa, North and South America, Asia, Europe, and other regions of the world [12]. Since its discovery, it has been reported that *V. parahaemolyticus* infection is associated with various serotypes or strains, with O1: K38, O3: K29, O4: K8, O3: K6, and O2: K3 being the prominent ones [10]. Although not all strains of this bacterium are considered pathogenic, those with virulent potential are usually differentiated from probably avirulent strains by the presence of genes encoding heat-stable direct hemolysin (TDH) and/or TDH-related hemolysin (TRH) [13]. In 1996, it was first identified that the O3: K6 strain expressed virulence genes. Subsequently, this serotype was isolated in various regions of the world, which led to its recognition as the first pandemic clone of *V. parahaemolyticus* identified and, to date, the most relevant globally [14].

*V. parahaemolyticus* is responsible for acute hepatopancreatic necrosis disease (AHPND) and translucent postlarval disease (TPD) in shrimp, two lethal pathologies that generate considerable economic losses in the aquaculture sector [15]. In humans, the clinical picture of infection with *V. parahaemolyticus* presents with watery diarrhea lasting between two and ten days, abdominal pain, nausea, vomiting, and headache, and cases of sepsis and wound infection have been reported, often fatal, in immunocompromised patients or with pre-existing liver disease or diabetes [6]. Although no unified global consensus exists on infection numbers, *V. parahaemolyticus* is estimated to cause approximately 35,000 human infections each year in the United States alone and has been the leading cause of foodborne infections in China since the 1990s [16].

Given the evidence of the cosmopolitan distribution of *V. parahaemolyticus*, it has been suggested that the appearance of epidemics is mainly associated with environmental determinants, especially climate change [17]. In that context, it has been reported that ocean temperatures have increased at an accelerated rate over the past two decades, a situation that directly favors the adaptability of the bacterium in ocean and coastal waters in different parts of the world [18]. Thus, while most climate events cannot be totally avoided, much of their health impacts can be mitigated by strengthening climate-resilient health systems, including effective risk reduction, preparedness, response, and recovery strategies [19]. In this article, we discuss the clinical and epidemiological aspects of human vibriosis caused by *V. parahaemolyticus*, a diarrheal disease of global relevance that, despite its importance, has received little public health attention. Given the complex interactions between environmental change, marine ecosystems, aquaculture practices, and human health, *V. parahaemolyticus* represents a paradigmatic One Health challenge. Despite the growing literature on *Vibrio parahaemolyticus*, evidence remains fragmented across clinical epidemiology, virulence, antimicrobial resistance, environmental ecology, climate-related drivers, and aquaculture-associated disease. Few reviews have integrated these dimensions within a unified One Health framework that simultaneously considers human health, aquatic animal health, food systems, and environmental change. This review addresses this gap by synthesizing these interconnected domains, with particular emphasis on how climate change influences pathogen distribution, the emergence of antimicrobial resistance, the burden of *V. parahaemolyticus* in shrimp and bivalve aquaculture, and the implications for surveillance, food safety, and sustainable disease prevention. By bringing these components together, this review offers an updated, interdisciplinary perspective on the evolving risks posed by *V. parahaemolyticus* in a changing world. Table 1 summarizes these interconnections. Figure 1 summarizes the major environmental, aquaculture, food-system, and human-health transmission pathways of *Vibrio parahaemolyticus*, along with key One Health mitigation interfaces.

## 2. Literature Search Strategy

We conducted a literature search in PubMed, Web of Science (WoS), Scopus, and SciELO to identify relevant publications on *Vibrio parahaemolyticus*. No publication date restrictions were applied, and the search included articles from database inception through 1 July 2026. Search terms were used individually and in different combinations and included: “*Vibrio parahaemolyticus*”, “vibriosis”, “epidemiology”, “prevalence”, “outbreak”, “foodborne infection”, “seafood”, “shellfish”, “oysters”, “shrimp”, “aquaculture”, “virulence”, “virulence factors”, “thermostable direct hemolysin”, “TDH”, “TDH-related hemolysin”, “TRH”, “type III secretion system”, “antimicrobial resistance”, “antibiotic resistance”, “biofilm”, “climate change”, “ocean warming”, “temperature”, “salinity”, “environmental reservoirs”, “acute hepatopancreatic necrosis disease”, “AHPND”, “translucent postlarval disease”, “TPD”, and “One Health”. We used Boolean operators (AND/OR) to combine terms according to the thematic domains addressed in the review. Publications were considered according to their relevance to the epidemiology, pathogenicity, antimicrobial resistance, environmental ecology, climate-related drivers, human disease, and aquaculture implications of *V. parahaemolyticus*. During manuscript preparation, the authors used ChatGPT Plus Images (OpenAI) to develop three original figures. The authors have reviewed and edited the output and take full responsibility for the content of this publication.

## 3. General Characteristics

*V. parahaemolyticus* is a Gram-negative, halophilic, thermolabile bacillus with ubiquitous distribution, considered the most prevalent foodborne pathogen associated with seafood consumption [20]. It belongs to the domain Bacteria, kingdom Pseudomonadati, phylum Pseudomonadota, class Gammaproteobacteria, order Vibrionales, family Vibrionaceae, genus *Vibrio* (NCBI Taxonomy: https://www.ncbi.nlm.nih.gov/datasets/taxonomy/670/, accessed 10 September 2026) [21]. Morphologically, it can present as a cell with a single polar flagellum or swarming motility, where it is provided with several lateral flagella [22].

Marine bacteria of the family Vibrionaceae are important for their role in the marine geochemical cycle, as well as symbionts and opportunistic pathogens of aquatic animals and humans [23]. These bacteria are characterized by their remarkable genomic, phylogenetic, and functional diversity, as well as a high sensitivity to physicochemical changes and a rapid replication rate, which allows them to respond effectively to environmental stimuli, such as those induced by climate change [24,25]. Its genome, typically composed of two chromosomes and multiple cryptic plasmids, exhibits remarkable plasticity, reflected in the abundance of genetic mobile elements such as transposons, prophages, and integrases [26,27]. This genomic structure indicates high genetic promiscuity. In this way, Vibrionaceae bacteria represent one of the most abundant bacterial groups in oceans, coastal areas, and inland water bodies [25].

The historical background of *V. parahaemolyticus* dates back approximately 70 years, when, between October 20 and 21, 1950, there was an outbreak of food poisoning in the Japanese cities of Osaka, Kishiwada, Kaizaka, and Izumi-Sano, associated with the consumption of shirasu (small fish fry, also known as white fish). In total, 272 cases of poisoning were reported, including 20 deaths. The incubation period was predominantly 2 to 6 h, although five patients presented symptoms within the first hour, and seven within 10 to 24 h after consumption. The main clinical symptoms were those characteristic of severe acute gastroenteritis: vomiting, diarrhea, severe abdominal pain, and moderate fever. Additionally, manifestations such as cooling of the limbs, cyanosis, dyspnea, chills, and joint pain were reported. Postmortem findings included mild erosions in the jejunum and ileum, catarrhal gastritis, and pulmonary interlobar hemorrhage in most cases, as well as pleural hyperemia and petechiae. Although several microorganisms were isolated, the researchers identified *Proteus morganii* and *Pasteurella parahaemolytica* as the most likely bacterial pathogens responsible for the clinical picture [11,28].

Later, in 1955, Iwao Takikawa isolated a similar bacterium in a separate case of food poisoning, which he named Pseudomonas enteritis [29]. Although the isolates resembled *Vibrio*, the cells’ straight shape differed from the genus’s characteristic curved morphology. In 1960, Davis and Parks contributed to the progress of taxonomy by defining the taxonomic position of the genus *Vibrio*, which allowed Fujino to re-examine the previously described isolates and confirm that they corresponded to the same species, which was named *V. parahaemolyticus* [30].

The bacterium *V. parahaemolyticus* is serotyped based on its heat-stable O-antigen polysaccharide (O antigen) and its capsular polysaccharide (K antigen), which are found on the bacterial surface, and is classified into 16 O serotypes and 71 K serotypes [31]. Researchers discovered the pandemic strain of *V. parahaemolyticus*, O3: K6, in Kolkata, India, in 1996 [32]. Shortly thereafter, other serotypes, such as O4: K68, O1: K25, and O1: KUT, were documented as exhibiting molecular characteristics identical to those of the O3: K6 serotype, so it is believed that these appeared to diverge from the O3: K6 serotype by altering O: K antigens and were defined as serovariants of the O3: K6 isolate [33,34].

To date, around 27 serovariants have been identified worldwide, with O1: K25, O1: K41, O1: K56, O1: KUT, O3: K58, O3: K68, O3: K75, O4: K8, O4: K12, O4: K68, O4: KUT, O5: KUT, and OUT: K6 being the most important after O3: K6 [35]. Through genetic sequencing, it has been possible to identify that, as with O3: K6, all pandemic serovariants share specific markers such as the presence of the thermostable direct hemolysin gene (*tdh*+), the absence, with exceptions, of the TDH-related hemolysin gene (*trh*−), and a distinctive toxRS sequence (toxRS/new), which are determinants of their ubiquity and enteropathogenicity [36].

## 4. Adaptability of *V. parahaemolyticus* to Climate Change

One of the repercussions of climate change is the increase in ocean temperatures, which favors the proliferation of pathogenic bacteria and the expansion of their distribution areas, driven by the loss of biodiversity and changes in environmental conditions [37]. The positive effect of seawater temperature on the abundance of *V. parahaemolyticus* has been observed in temperate regions with low and moderate temperatures and a clear seasonal pattern, such as North America and Europe [38]. Conversely, seawater temperature has been shown to have a minimal influence on *Vibrio* abundance in tropical areas, where water temperature values remain almost constant throughout the year [39]. In terms of seasonality, the highest incidence rates of *V. parahaemolyticus* in fish and humans have been recorded in September and July [17,40].

Climate change and warming oceans have a major impact on microbial communities, leading to an increased prevalence of *V. parahaemolyticus* infections worldwide, including Alaska, where isolates of the bacterium have been reported [41,42]. Relatively small increases in temperature (3 °C) in intertidal ecosystems can generate significant changes in the microbiomes of aquatic fauna, such as oysters, favoring bacterial colonization by this pathogen and the consequent mortality of mollusks [43,44].

The influence of climate change on marine bacteria is not limited to thermal increase, but also includes a decrease in salinity, increased acidification, alterations in ocean circulation patterns, and sea level rise [45]. V. *parahaemolyticus* survives in environments containing 0.5% NaCl, and the bacterium responds to osmotic stress through osmoregulation and the T3SS1 and T3SS2 secretion systems, which induce cytotoxicity and a gastroenteritis response. Thus, low-salinity environments can result in high pathogenicity of *V. parahaemolyticus* [46].

## 5. Virulence Factors

The pathogen *V. parahaemolyticus* is distinguished by its ability to spread globally and cause significant damage and injury to aquatic animals and humans [7]. For more than 50 years, the bacterium has been known to possess virulence factors that facilitate its pathogenicity and cytotoxicity [47]. To date, at least eight virulence factors associated with the enteropathogenicity of *V. parahaemolyticus* have been described, namely: hemolysins (the most relevant), type III secretion system (SST3), type 6 secretion system (SST6), adhesion factors, iron uptake system, lipopolysaccharides (LPS), proteases, and outer membrane proteins (OMPs) [48]. Table 2 summarizes the main virulence determinants, their genetic basis, and mechanisms of action.

The first virulence factor of *V. parahaemolyticus* was discovered in 1969, when a hemolysin produced by the bacterium was reported to cause β-type hemolysis on Wagatsuma agar, which became known as the “Kanagawa phenomenon.” [49]. Hemolysins are crucial for this pathogen, so much so that, to identify *V. parahaemolyticus*, the U.S. Food and Drug Administration’s (FDA) Manual of Bacteriological Analysis (BAM) recommends a polymerase chain reaction (PCR) protocol to detect the species-specific thermostable hemolysin (tlh) gene [50].

Thermostable direct hemolysin (TDH), encoded by the tdh gene, and TDH-related hemolysins (TRH), encoded by the TRH gene, are the major virulence factors expressed by *V. parahaemolyticus* [51]. Accordingly, a study found that more than 80% of clinical isolates of *V. parahaemolyticus* are positive for the TDH gene. In contrast, fewer than 20% are positive for the TRH gene. In turn, more than 90% of patients with infections caused by this pathogen have been positive for TDH [52]. In contrast, isolates from environmental sources are generally considered avirulent to humans, as only a small proportion are positive for these genes [53]. Regarding isolates from aquatic animals, 1.57% of *V. parahaemolyticus* isolates from shellfish were positive for TDH and 3.66% for TRH. In a study of infected marine mussels, TDH was detected in 9.8%, TRH in 0.5%, and 1.5% had both genes [54]. This highlights that aquatic products, such as shellfish and mussels, are an important source of human infection, as *V. parahaemolyticus* is recognized as an aquatic zoonotic pathogen of global relevance [55].

From a structural point of view, TDH is a pore-forming toxin (PFT) that alters the plasma membrane of erythrocytes, allowing the passage of ions and water, leading to erythrocyte lysis and causing potent cytotoxicity; it also stimulates the secretion of chlorides in the intestinal epithelium by means of an intracellular increase in Ca^2+^, triggering watery diarrhea [12,56,57]. TRH is a thermolabile toxin composed of 189 amino acids and exhibits biological activities similar to those of TDH [12]. The TRH gene, a precursor to the TRH toxin, is thought to contribute to the pathogenicity of *V. parahaemolyticus* and promote its survival after entering the human body, as it is associated with the ure gene, which stimulates the production of urease, an enzyme that catalyzes the hydrolysis of urea into ammonia, which raises the pH of the environment within the host.

## 6. Epidemiology

The emergence of the pandemic serovar O3: K6 in 1996 has facilitated the distribution of *V. parahaemolyticus* in different coastal and estuarine regions of the world [34]. Although its distribution is transcontinental, the most affected countries are China and the United States [58,59]. However, there is evidence of isolates in Argentina, Australia, Bangladesh, Brazil, Chile, Colombia, France, Germany, Mexico, Panama, Peru, South Korea, and other countries around the world, highlighting the adaptability of *V. parahaemolyticus* to different environments [34,60,61,62,63]. Table 3 summarizes major outbreaks and key epidemiological characteristics across regions.

In China, *V. parahaemolyticus* is a leading cause of foodborne bacterial gastroenteritis, characterized by marked seasonality and high prevalence in seafood. Between 2010 and 2022, Zhejiang province reported 383 outbreaks associated with this pathogen, resulting in 4382 cases of illness, 326 hospitalizations, and 1 death. Predominant symptoms included diarrhea (95, 18%), abdominal pain (89, 23%), nausea (55, 64%), vomiting (50, 57%), and fever (24, 21%). In total, 77.54% of outbreaks occurred in July to September, coinciding with the seasonal increase in temperature and seafood consumption. Regarding the place of occurrence, 57.96% of the outbreaks were registered in restaurants, followed by staff canteens (15.40%) and rural banquets (11.23%). Additionally, 31.85% of outbreaks were related to the consumption of aquatic products, while 12.53% were linked to ready-to-eat foods, such as traditional Chinese cold dishes and cooked meats [64].

Between 2000 and 2009, a study in eleven Chinese provinces detected the pathogen in 3.11% of 79,075 fecal samples from patients with acute diarrhea, which evidenced their role as a significant pathogen in coastal areas of the country [65]. Surveillance in the southern coastal region of China (Shenzhen and adjacent provinces) between 2007 and 2012 confirmed that *V. parahaemolyticus* was the most common etiological agent in bacterial diarrhea outbreaks, with the pandemic serotype O3: K6 predominant, followed by O4: K8 and O3: K29 [66]. A study conducted in eastern China (2009 to 2013) examined 6951 fecal samples from patients with acute diarrhea, of which *V. parahaemolyticus* was isolated in 563 cases (approximately 8.1%). Positivity peaked from June to August, with a detection rate of 13.1%, while in winter it barely reached 0.2%. In 89% of positive samples, *V. parahaemolyticus* was the only agent identified [67].

Regarding the pathogen’s presence in marine products, multiple studies have documented high contamination rates in seafood. Between 2017 and 2019, out of 900 samples tested in Chinese markets, contamination rates were 15.34% in shrimp, 10.67% in fish, and 3.67% in ready-to-eat foods, with a total of 101 isolates [68]. In retail fish, shrimp, and ready-to-eat foods (905 samples), 11.16% were positive for *V. parahaemolyticus*, and in summer, this prevalence was almost double that observed in winter (14.87% vs. 7.26%). A total of 202 strains were isolated, of which 9.90% carried the TDH gene, and 19.80% carried the TRH gene [69]. Another study conducted on the northern coast of China revealed that 23.4% and 43.4% of fish and shrimp samples from retail seafood stores contained *V. parahaemolyticus* [70].

In the United States, the pandemic serotype (O3:K6) was first reported in 1998, in association with the largest outbreak of *V. parahaemolyticus* in the country’s history, with higher incidence reported in the Pacific Northwest region [60]. In 1988 and 1997, 345 sporadic infections with *V. parahaemolyticus* were reported, of which 59% manifested as gastroenteritis, 34% as wounds, 5% as sepsis, and 2% were due to other exposures; 45% of patients who suffered from these conditions were hospitalized for their infections, and 88% of people with acute gastroenteritis reported eating raw oysters during the week before their illness occurred [71]. Environmental isolates of *V. parahaemolyticus* have been found in water and aquatic animals such as oysters and crabs across different coastal regions of the United States [72,73,74].

In Europe, the epidemiology of *V. parahaemolyticus* extends beyond France and includes both sporadic infections and well-documented outbreaks across several countries [75]. Spain, particularly Galicia, represents an important European hotspot: in 1999, an outbreak associated with the consumption of raw oysters caused 64 cases of acute gastroenteritis, including nine hospitalizations, and pathogenic *tdh*-positive strains were isolated. A larger seafood-associated outbreak occurred in Galicia in 2012, involving 100 of 114 passengers on a cruise boat, with shrimp identified as the most probable source; isolates included virulent strains genetically related to Pacific Northwest lineages. In northern Europe, an exceptionally warm summer in 2018 was associated with 445 non-travel-related vibriosis cases in Norway, Sweden, Denmark, Finland, Poland, and Estonia, of which 89 (20.0%) were caused by *V. parahaemolyticus*; 87.6% of these infections occurred during summer, with cases concentrated particularly around the North Sea and connecting Baltic Sea coastal areas. Clinical and environmental *V. parahaemolyticus* have also been documented in the United Kingdom, Italy, France, and Germany, reinforcing evidence that this pathogen is established across European marine environments and that its public-health importance may increase with warming coastal waters [76,77,78,79,80,81].

In Central America, researchers first detected *V. parahaemolyticus* in Panama’s marine waters in 1974 [82]. A year later, the country confirmed the first case of gastroenteritis associated with this pathogen. Researchers isolated the bacterium from both the feces of a patient with acute gastroenteritis and fresh shrimp. This case was linked to an outbreak that severely affected 39 other people, all of whom developed symptoms within hours of consuming shrimp at the same restaurant [83]. Around 1980, in Mexico, a healthy child developed *V. parahaemolyticus* endophthalmitis after suffering a corneal perforation while swimming in the Gulf of Mexico, so they recommended that a history of eye injury in a saltwater environment should alert the doctor to the possibility of infection by this marine bacterium [84]. Likewise, surveillance of 144 clinical strains of *V. parahaemolyticus* from 2004 to 2011 found that 66% of isolates belonged to the pandemic clone O3: K6, of which 26.3% were TDH+ and/or TRH+ [85].

The coasts of South America have also been a conducive environment for the presence of *V. parahaemolyticus* [86]. In Colombia, 17 isolates have been reported, of which seven correspond to clinical cases and ten to environmental sources [87]. In Brazil, the pathogen has been isolated in the context of diarrhea outbreaks, as well as in oysters and seawater samples [88,89]. Similarly, *V. parahaemolyticus* was first detected in Argentina in 1977, from farmed mussels [90].

The epidemiology of *V. parahaemolyticus* in countries such as Chile has been the subject of intense study since the country began to experience recurrent outbreaks of gastroenteritis associated with the consumption of contaminated seafood [91]. Since 1998, Chile has experienced multiple epidemic episodes caused by strains of *V. parahaemolyticus*, mainly the pandemic clone O3:K6. One of the most intense outbreaks occurred in 2005 in the city of Puerto Montt, where more than 3 outbreaks were reported. A total of 600 cases of acute diarrhea were attributed to this pandemic strain [92]. Despite control efforts, this clone persisted in later years, accounting for 73% of the cases recorded in the 2007 outbreak, when 475 clinical cases were reported in the same region [93].

At the same time, researchers have investigated the pathogen’s presence in marine environments and seafood products, revealing an alarming frequency of contamination. During the austral summer of 2004–2005, *V. parahaemolyticus* was detected in 53% of shellfish samples collected in culture and extraction areas, with concentrations ranging from 3 to 93 units per gram of meat. Although only a small percentage (6%) of these environmental isolates corresponded to the pandemic clone O3:K6, the non-pandemic strains showed remarkable genetic diversity, distributed across at least 14 distinct groups based on pulsed-field electrophoresis patterns [94].

In relation to marine fauna, the presence of *V. parahaemolyticus* has been documented in several species of bivalve mollusks, including mussels and clams [95]. In a study conducted in southern Chile during the 2006 outbreak, 80% of the shellfish sampled contained pathogen strains, of which the tdh-positive ones corresponded exclusively to the O3: K6 clone. The remaining, tdh-negative, were grouped into at least six distinct groups, indicating a dynamic bacterial ecosystem with pathogenic potential [96].

In addition, a previous study reported that, between 1997 and 1998, 19 of 20 clinical strains isolated in Antofagasta also belonged to the pandemic clone, confirming their geographical spread along the Chilean coast even before the largest outbreaks [97]. This dispersal coincided with rising sea surface temperatures, suggesting a link between climate change and the proliferation of virulent V. parahaemolyticus strains in temperate marine zones [98].

In 2009, researchers detected a new and extensive outbreak of *V. parahaemolyticus* in Peru. Between February and March of that year, researchers obtained 30 isolates from clinical samples of patients with gastroenteritis in Lima, Cajamarca, Chiclayo, and Piura. All strains belonged to serotype O3: K59, a serotype not previously identified in Peru. In addition, all were positive for TDH and negative for TRH [99]. In contrast, another study reported that 94.1% and 5.9% of clinical isolates expressed TDH and TRH, respectively [100]. On the other hand, in shrimp farmed in the northern region of Peru, nine strains of *V. parahaemolyticus* were isolated that encoded genes similar to pirAB, representing a possible risk factor for the occurrence of APHND outbreaks [101].

## 7. Clinical Management and Treatment

Most *V. parahaemolyticus* gastroenteritis is self-limited, and management is primarily supportive, with oral rehydration and correction of fluid and electrolyte losses; intravenous fluids may be required in patients with severe dehydration or inability to tolerate oral intake. Routine antimicrobial therapy is not recommended for mild, uncomplicated gastroenteritis. However, consider antibiotics in severe or prolonged disease, and use them for invasive infections, including bacteremia, sepsis, and clinically significant wound infections. Doxycycline is commonly recommended for severe *V. parahaemolyticus* gastroenteritis, while guidance for invasive non-cholera *Vibrio* infections includes a third-generation cephalosporin, such as ceftriaxone, combined with doxycycline; antimicrobial selection should subsequently be guided by culture and susceptibility testing, particularly given emerging resistance [102]. Wound infections also require careful local management, and severe cases may require surgical debridement of necrotic tissue. Early recognition and prompt treatment are especially important in patients with underlying liver disease, immunosuppression, diabetes, or other conditions associated with severe vibriosis [103,104,105,106].

## 8. Antimicrobial Resistance

The extensive use of antibiotics in aquaculture has resulted in the emergence of antibiotic-resistant strains in the environment [107]. Although fluoroquinolones and third-generation cephalosporins generally retain activity against *V. parahaemolyticus*, reduced susceptibility and resistant isolates, including resistance to ciprofloxacin and ceftriaxone, have increasingly been reported [108]. The ability of this pathogen to resist antibiotics is known to be mediated by biofilm formation and the expression of specific genes [109]. Restricting antimicrobial use in aquaculture to clearly indicated situations, under veterinary oversight and antimicrobial-stewardship principles, is therefore essential to reduce selective pressure and limit the emergence and dissemination of resistant *V. parahaemolyticus* strains. Table 4 summarizes the main antimicrobial resistance patterns, mechanisms, and their One Health implications.

A biofilm is a community of microorganisms attached to a surface, embedded and growing in a self-produced matrix of extracellular polymeric substances, which can resist adverse conditions and lead to persistent infection [110]. *V. parahaemolyticus* is usually found forming biofilms. The development of the biofilm follows a cycle that begins with cell adhesion, continues with structured growth, and culminates with the release of cells into the environment [109]. The biofilms formed by *V. parahaemolyticus* act as a protective barrier against antibiotics by encapsulating bacterial cells within an extracellular matrix (EPS) that limits drug penetration and facilitates bacterial persistence in harsh environments [111].

*V. parahaemolyticus* can express up to ninety-four antibiotic resistance genes; some of them are BLA (beta-lactams), TET (tetracyclines), Sul2 (sulfonamide), and FloR (chloramphenicol) [112]. In a study published in 2024, it was reported that 19.1% of *V. parahaemolyticus* isolates had acquired resistance to at least one class of antibiotic, with multidrug-resistant strains also being found [113]. Susceptibility patterns vary among studies and isolate sources. In one study, *V. parahaemolyticus* isolates showed high resistance to ampicillin, erythromycin, tetracycline, sulfisoxazole, rifampicin, cefazolin, cephalothin, and streptomycin, while remaining susceptible to chloramphenicol, ceftriaxone, ciprofloxacin, gentamicin, and nalidixic acid [114].

## 9. Biofilm Formation and Persistence

*Vibrio parahaemolyticus* readily forms biofilms on biotic and abiotic surfaces, a trait that contributes importantly to its persistence in marine environments, aquaculture systems, seafood, and host-associated settings. Biofilm development involves initial surface attachment, maturation within a self-produced extracellular polymeric substance (EPS) matrix, and subsequent dispersal of bacterial cells. This organized structure enhances bacterial survival under unfavorable environmental conditions and may facilitate prolonged persistence and dissemination [115,116].

Beyond environmental survival, biofilm formation has important implications for virulence and disease control. The EPS matrix can reduce antimicrobial penetration and increase bacterial tolerance to antibiotics, thereby contributing to treatment failure and the persistence of resistant populations. Biofilms may also favor bacterial retention on aquaculture equipment, tanks, processing surfaces, and seafood-associated substrates, complicating sanitation and facilitating recurrent contamination. Consequently, strategies to prevent surface colonization, improve cleaning and disinfection, and disrupt established biofilms should be considered an important component of *V. parahaemolyticus* control across aquaculture, food-processing, and clinical interfaces [117,118].

## 10. Acute Hepatopancreatic Necrosis Disease (AHPND)

Shrimp aquaculture is the fastest-growing sector within the fishing industry, with a crucial role in the global economy, employment, and food supply. Its expansion, driven by high demand, has made this activity an especially significant export industry in Southeast Asia, with annual revenues of billions of dollars [119]. Global shrimp production is geographically concentrated, particularly across Asia and Latin America, although production occurs across multiple regions worldwide (Figure 2). At the farm level, shrimp health and production are shaped by interconnected environmental, anthropogenic, biological, and socio-economic factors, as well as management practices and supply-chain pressures. Figure 3 summarizes the relationships among these factors, key stressors, disease risks, resilience strategies, and broader sustainability outcomes. However, the intensification of cultivation has brought with it serious challenges, with infectious diseases being the main limiting factor, causing cumulative losses of close to 15 billion dollars over the last fifteen years [120]. Currently, acute hepatopancreatic necrosis disease caused by *V. parahaemolyticus* represents the most serious threat due to the disease’s perpetuation over time and the associated economic losses [121]. Between 2009 and 2016, China, Thailand, and Mexico reported, respectively, losses of $11 billion, $8 billion, and about $1 billion from AHPND [122].

In 2009, an emerging disease was reported in shrimp that was initially called early mortality syndrome (EMS) because it causes massive early mortality in shrimp ponds, reaching up to 100% in a few days after infection; in 2011, the name acute hepatopancreatic necrosis syndrome (AHPNS) was proposed, classified as an idiopathic disease because no causative agent had been identified [123]. In early 2013, at the University of Arizona’s Aquaculture Pathology Laboratory, *V. parahaemolyticus* was isolated and identified as the causative agent, and the condition was renamed from syndrome to disease [124]. The pathogenicity of the AHPND-causing *V. parahaemolyticus* strains is mainly attributed to the presence of the plasmid pVA, which encodes two homologous toxin genes, pyrA and pirB [125].

According to FAO, to date, AHPND remains the most economically important bacterial disease affecting both tiger shrimp (*Penaeus monodon*) and Pacific white shrimp (*Penaeus vannamei*) [126]. However, other penaeids, such as the Kuruma shrimp (*P. japonicus*) and the Chinese white shrimp (*P. chinensis*), are also susceptible [127]. Shrimp affected by AHPND present with lethargy, anorexia, slow growth, and an empty digestive tract, and the disease is characterized by severe atrophy of the hepatopancreas and a mortality rate of up to 100% within the first 35 days of the postlarval stage [128]. However, these clinical signs are also commonly observed in other shrimp diseases, such as necrotizing hepatopancreatitis (NHP-B) and septic hepatopancreatic necrosis (SHPN), which is why the diagnosis of AHPND should include the histopathological lesions observed in the acute phase of the disease and, mainly, the molecular detection of the pyrA and pirB genes [125,129].

Although the World Organization for Animal Health (WOAH/OIE) recognized it as a notifiable aquatic animal disease in 2016, it remains difficult to achieve a comprehensive management and control system for AHPND [130]. This is due to variations in water temperature, osmotic stress, and antibiotic resistance resulting from the extensive use of antimicrobials [131]. Consequently, studies are currently underway on bacteriophages as biocontrol agents, β-glucans derived from marine diatoms as immunostimulants, and genetic improvement to produce shrimp with higher body weight, to counteract the health and economic impact of AHPND on shrimp [132,133].

## 11. Translucent Postlarval Disease (TPD)

From late 2019 to early 2020, a new disease was recorded in the shrimp *Penaeus vannamei* called translucent postlarval disease (TPD) [134]. Also called glass postlarvae disease, TPD was first reported in China’s Fujian and Guangdong provinces in late 2019 [135]. Between April and May 2020, TPD spread rapidly across major shrimp farming areas in China, causing up to 80% of hatcheries in major coastal regions to suspend production of *Penaeus vannamei* postlarvae [136].

Although TPD was initially believed to be caused by a novel virus, research showed that three highly lethal strains of *V. parahaemolyticus*, vp-HL-201910, vp-HL-202005, and vp-HL-202006, are involved in the etiology of the disease [135]. At first, it was deduced that AHPND *Vibrio* (*Vp* AHPND) was the same causative agent of TPD (*Vp* TPD) because the macroscopic clinical signs of diseased postlarvae were similar. However, AHPND occurs approximately 35 days after shrimp pond stocking, and clinical signs and mortality begin approximately 10 days after stocking [137]. In contrast, TPD usually occurs in postlarvae 4 to 7 days later, much earlier than AHPND usually appears [137]. In addition, *Vp* AHPND expresses the pyrA and pirB genes, while TPD expresses the vhvp-2 gene [138,139].

Infection with *Vp TPD* has a severe detrimental effect on the liver, pancreas, and midgut epithelial cells of shrimp, leading to significant tissue damage [134]. Characteristic macroscopic clinical signs include anorexia, slow growth, decreased swimming capacity, an empty digestive tract, and a pale or colorless hepatopancreas, resulting in a translucent appearance of the diseased animals that gives the disease its name [137]. Cumulative mortality of infected postlarvae could reach 100% within 3 days in a typical case. Likewise, the toxicity of *Vp* TPD to *P. vannamei* postlarvae has been reported to be 1000 times greater than that of *Vp* AHPND [136,140].

## 12. Recent Findings About Controlling *V. parahaemolyticus* Infections in *P. vannamei*

### 12.1. Inheritance Resistance Trait

New research to control vibriosis in *P. vannamei* shrimp includes understanding the defense mechanism of colonization resistance against vibriosis and the inheritance of the resistance trait [141]. They constructed vibriosis-resistant and susceptible families after four generations of artificial selection. Microbiome sequencing indicated that shrimp can successfully develop a colonization resistance trait against *Vibrio* infections. This trait was characterized by a microbial community structure with specific enrichment of a single probiotic species (namely *Shewanella algae*), and notably, it was heritable. The host may memorize it through epigenetic remodeling. Regardless of the infection status, a group of genes was specifically activated in the resistant family through disruption of complete methylation. Specifically, hypomethylation and overexpression of genes related to lactate dehydrogenase (LDH) and iron homeostasis may provide rich sources of carbon (lactate) and ions for *S. algae* colonization, which directly reduces *Vibrio* load in shrimp. Lactate feeding increased the survival of shrimp, while knockdown of the LDH gene decreased survival when *Vibrio* pathogens infected shrimp. In addition, treatment of shrimp with the methyltransferase inhibitor 5-azacytidine resulted in upregulation of LDH and some protein processing genes, significant enrichment of *S. algae*, and simultaneous reduction in *Vibrio* in shrimp. Their results suggest that the host can remember colonization resistance as epigenetic information, which has played a pivotal role in vibriosis resistance. This information will aid in disease control and the selection of superior lines of shrimp with high disease resistance [141].

### 12.2. Regulation of ETosis in P. vannamei

Recently, researchers discovered bioactive compounds in FD-LAB that possess antibacterial and anti-virulence mechanisms against *V. parahaemolyticus* D6 by interfering with the pathogen recognition step and inhibiting the growth and virulence of *V. parahaemolyticus* D6, thereby preventing the pathogen from inducing an immune response in the host. These discoveries were obtained using an ex vivo approach; subsequent in vivo studies would further elucidate the effects of FD-LAB on shrimp ETosis and optimize its use in practical settings. The study also provided crucial information on the role of interfering with ROS production in ETosis regulation. Further studies should characterize other potential mechanisms of action of FD-LAB on ETosis pathways in shrimp and explore its potential application to regulating ETosis in other species [142].

Substantial barriers remain to achieving globally resilient shrimp aquaculture, and meaningful progress will require coordinated action at multiple levels. Policy support, sustainable financing, and implementation-focused research and development are essential to promote more resilient and responsible production systems while strengthening and empowering farming communities. Increasing productivity alone will be insufficient to meet future food demands sustainably. Instead, long-term resilience will depend on coordinated engagement across the entire shrimp value chain, including feed producers, farmers, processors, retailers, food service industries, governments, and non-governmental organizations. Integrating complementary solutions could transform how shrimp farms are designed, managed, financed, and monitored, while facilitating the development of locally adapted production models. Further research is needed to determine how to effectively incentivize and implement such approaches across diverse settings, particularly among small-scale and resource-constrained producers. Cross-disciplinary networks linking public and private stakeholders could help farmers co-develop practical, scalable interventions for immediate and medium-term use. The integration of bottom-up and top-down strategies may ultimately provide a more resilient framework for global shrimp aquaculture, while generating lessons applicable to other aquaculture systems [143].

## 13. Integrated One Health Strategies for Prevention, Surveillance, and Control of *Vibrio parahaemolyticus*

Mitigating the growing impact of *V. parahaemolyticus* requires coordinated interventions across human, animal, environmental, and food-production systems. From a One Health perspective, surveillance should integrate clinical cases, seafood contamination, aquaculture health, antimicrobial resistance, and environmental variables such as seawater temperature, salinity, and water quality. Such integration may facilitate earlier detection of changes in pathogen abundance, virulence, geographic distribution, and seasonal transmission patterns [144,145,146].

At the human–food interface, prevention should prioritize safe seafood handling, appropriate cooking, cold-chain maintenance, and microbiological surveillance of high-risk products, particularly raw or undercooked shellfish. In aquaculture, strengthened biosecurity, improved water-quality management, responsible antimicrobial use, disease-free stocks, and early pathogen detection are essential to reduce disease emergence and economic losses. Environmental monitoring should complement these measures by identifying conditions that favor *Vibrio* proliferation, particularly in coastal areas affected by warming, altered salinity, pollution, and ecosystem degradation. Finally, coordinated data sharing among public health, veterinary, aquaculture, food-safety, and environmental authorities is essential for timely risk assessment and response. Together, these measures provide a practical One Health framework for reducing the public-health, animal-health, and food-security impacts of *V. parahaemolyticus* [63,86,117,147].

### 13.1. Climate-Sensitive Surveillance and Molecular Detection

Climate-driven changes, particularly rising seawater temperatures, altered salinity, extreme weather events, and deterioration of coastal water quality, can modify the abundance, seasonality, and geographic distribution of *V. parahaemolyticus*. Surveillance strategies should therefore integrate environmental indicators with continuous monitoring of aquaculture systems, seafood contamination, human cases, and antimicrobial resistance, particularly during high-risk seasons and in coastal areas with favorable environmental conditions. Laboratory surveillance should combine conventional microbiological methods with molecular tools. Polymerase chain reaction (PCR) targeting the species-specific *tlh* gene can support identification of *V. parahaemolyticus*, while detection of *tdh* and *trh* provides additional information regarding pathogenic potential; in shrimp aquaculture, molecular detection of *pirAB* genes is particularly relevant for identifying AHPND-associated strains. Harmonized sampling, laboratory procedures, and reporting systems would improve comparability among regions and facilitate earlier detection of emerging risks [54,147,148,149].

### 13.2. Seafood Safety and Aquaculture Biosecurity Across the Production Chain

Prevention should extend from pre-stocking aquaculture management to post-harvest processing and final consumption. Before stocking, measures should include appropriate water-quality management, use of healthy or pathogen-screened stocks, adequate stocking densities, farm hygiene, and measures to minimize pathogen introduction and amplification. During production, routine health surveillance, responsible antimicrobial use, and early disease detection should be prioritized. After harvest, risk-based microbiological screening of seafood, particularly shellfish and products intended for raw or minimally cooked consumption, should be combined with hygienic processing, rapid temperature control, cold-chain maintenance, cross-contamination prevention, and adequate cooking. Implementation of hazard analysis and critical control point (HACCP)-based procedures throughout harvesting, processing, transport, storage, and food service can further reduce opportunities for bacterial proliferation and human exposure [117,150,151,152].

### 13.3. Governance, Trade, and Cross-Sectoral One Health Coordination

Effective control also requires coordinated governance across public health, veterinary, aquaculture, food-safety, environmental, and trade authorities. Risk-based national and international policies should promote harmonized microbiological standards, seafood traceability, certification and sanitary requirements, appropriate transport and storage conditions, and rapid information exchange when contaminated products or outbreaks are identified. International seafood trade may disseminate contaminated products across borders; therefore, surveillance and regulatory measures should be proportionate to risk and supported by transparent reporting and laboratory evidence. Integrating environmental monitoring, aquaculture surveillance, food-safety data, clinical notifications, and antimicrobial-resistance information would strengthen early warning and enable targeted interventions while supporting both public health and sustainable seafood production [151,153,154].

## 14. Limitations

This review has several limitations that should be acknowledged. First, as a narrative review, it does not follow a formal systematic methodology; therefore, study selection may be subject to selection bias and may not capture all available evidence on *Vibrio parahaemolyticus*. Although efforts were made to include relevant studies, the recent literature from multiple regions, and some geographic areas, particularly low- and middle-income countries, remain underrepresented because of limited surveillance data and publication gaps.

Second, heterogeneity in study design, laboratory methods, and case definitions limits direct comparisons across regions and time periods. Variability in diagnostic approaches, including differences in molecular detection of virulence genes (e.g., TDH and TRH), may influence reported prevalence and pathogenicity estimates. Additionally, reliance on secondary data limits the ability to assess causality, especially regarding the complex relationships among climate change, environmental factors, and the emergence of vibriosis.

Third, data on antimicrobial resistance patterns and aquaculture-related outbreaks are unevenly reported, which may lead to an incomplete understanding of the current global burden and trends. Emerging conditions such as acute hepatopancreatic necrosis disease (AHPND) and translucent postlarval disease (TPD) are still evolving fields, with limited longitudinal and standardized data available.

Finally, despite adopting a One Health perspective, integration across human, animal, and environmental health domains remains constrained by fragmented data systems. Strengthening interdisciplinary surveillance and standardized reporting frameworks will be essential to understand this pathogen better.

## 15. Conclusions

*Vibrio parahaemolyticus* has emerged as a globally significant pathogen at the intersection of human health, marine ecosystems, and food production systems. Its expanding distribution, driven by ocean warming, environmental change, and aquaculture intensification, underscores the growing relevance of this bacterium within a One Health framework. Beyond its well-recognized role in foodborne gastroenteritis, its impact on aquatic animal health, particularly through shellfish (shrimp) diseases such as AHPND and TPD, and mollusks (oyster) diseases, highlights important economic and food security implications [141,155].

The increasing detection of virulent strains, coupled with the emergence of antimicrobial resistance, raises additional concerns for clinical management and public health preparedness. At the same time, the complex interactions between environmental variables, microbial adaptation, and human exposure pathways complicate risk prediction and control strategies.

Addressing these challenges requires translating the One Health framework into clearly defined research, policy, and operational priorities. Priority research areas should include standardized multicountry surveillance, longitudinal studies linking climatic and environmental variables with human and aquaculture infections, molecular and genomic monitoring of virulence and antimicrobial-resistance determinants, validation of rapid diagnostic approaches, evaluation of non-antibiotic disease-control strategies in aquaculture, and predictive models capable of identifying emerging geographic and seasonal risks. From a policy perspective, greater integration of human, aquatic animal, food-safety, and environmental surveillance systems is needed, together with antimicrobial-stewardship programs, harmonized microbiological standards, seafood traceability, and climate-informed early-warning systems. Practical interventions should include stronger farm biosecurity, screening of stocks and high-risk seafood products, improved water-quality management, responsible antimicrobial use, cold-chain maintenance, HACCP-based processing, prevention of cross-contamination, and timely clinical recognition and management of severe infections. Implementing these coordinated measures will be essential to reduce the evolving public-health, aquaculture, and food-security impacts of *V. parahaemolyticus* in a changing global environment.

## Figures and Tables

**Figure 1:**
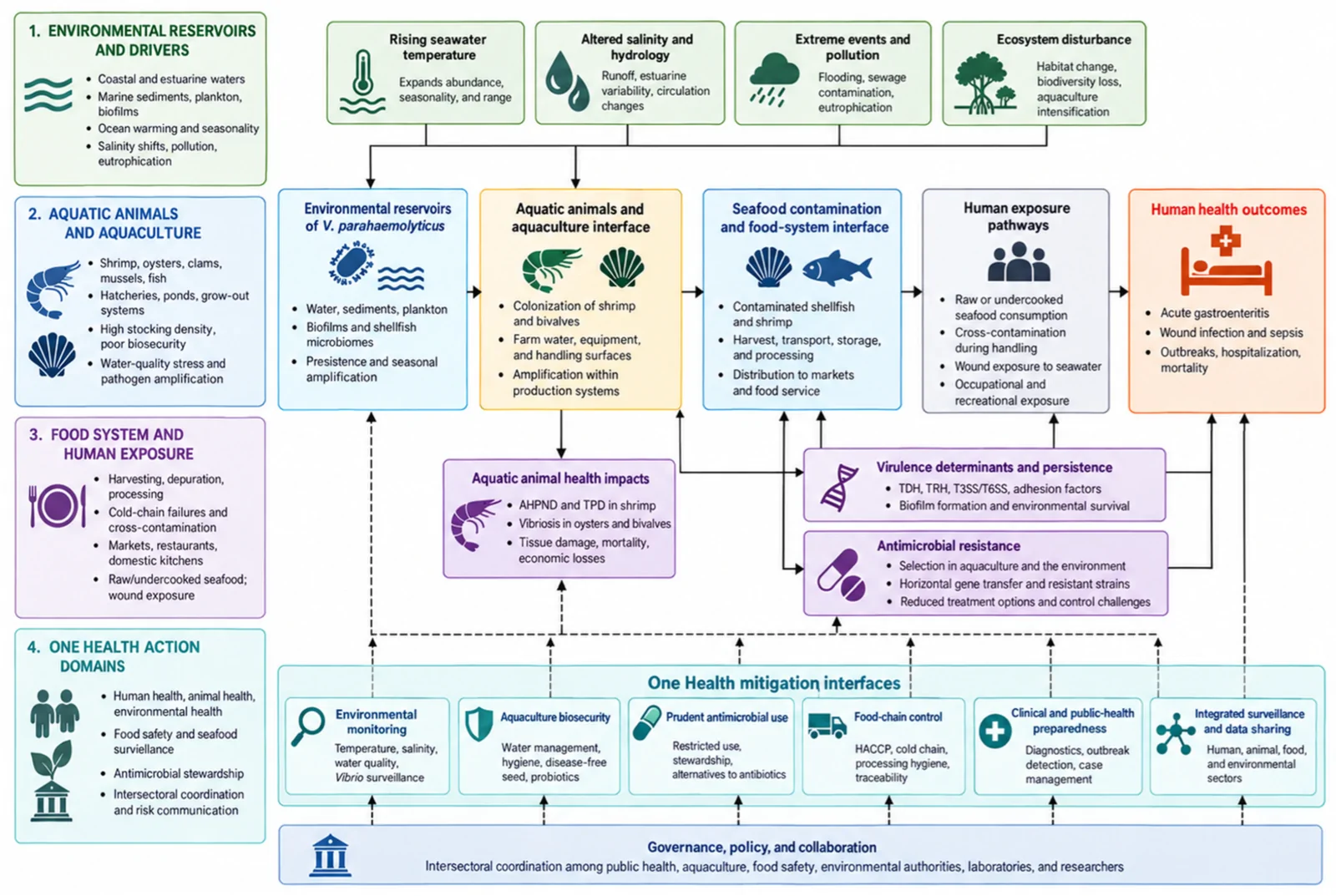
One Health transmission pathways and mitigation interfaces of *Vibrio parahaemolyticus*. Legend: → main transmission pathway, ---> mitigation/feedback. Abbreviations: AHPND, acute hepatopancreatic necrosis disease; HACCP, hazard analysis and critical control points; T3SS, type III secretion system; T6SS, type VI secretion system; TDH, thermostable direct hemolysin; TPD, translucent postlarval disease; TRH, TDH-related hemolysin; *V. parahaemolyticus*, *Vibrio parahaemolyticus*. Original figure created by the authors with AI-assisted image generation using ChatGPT Plus Images, 5.6 Sol (OpenAI), followed by author review and verification of all scientific content and terminology.

**Figure 2:**
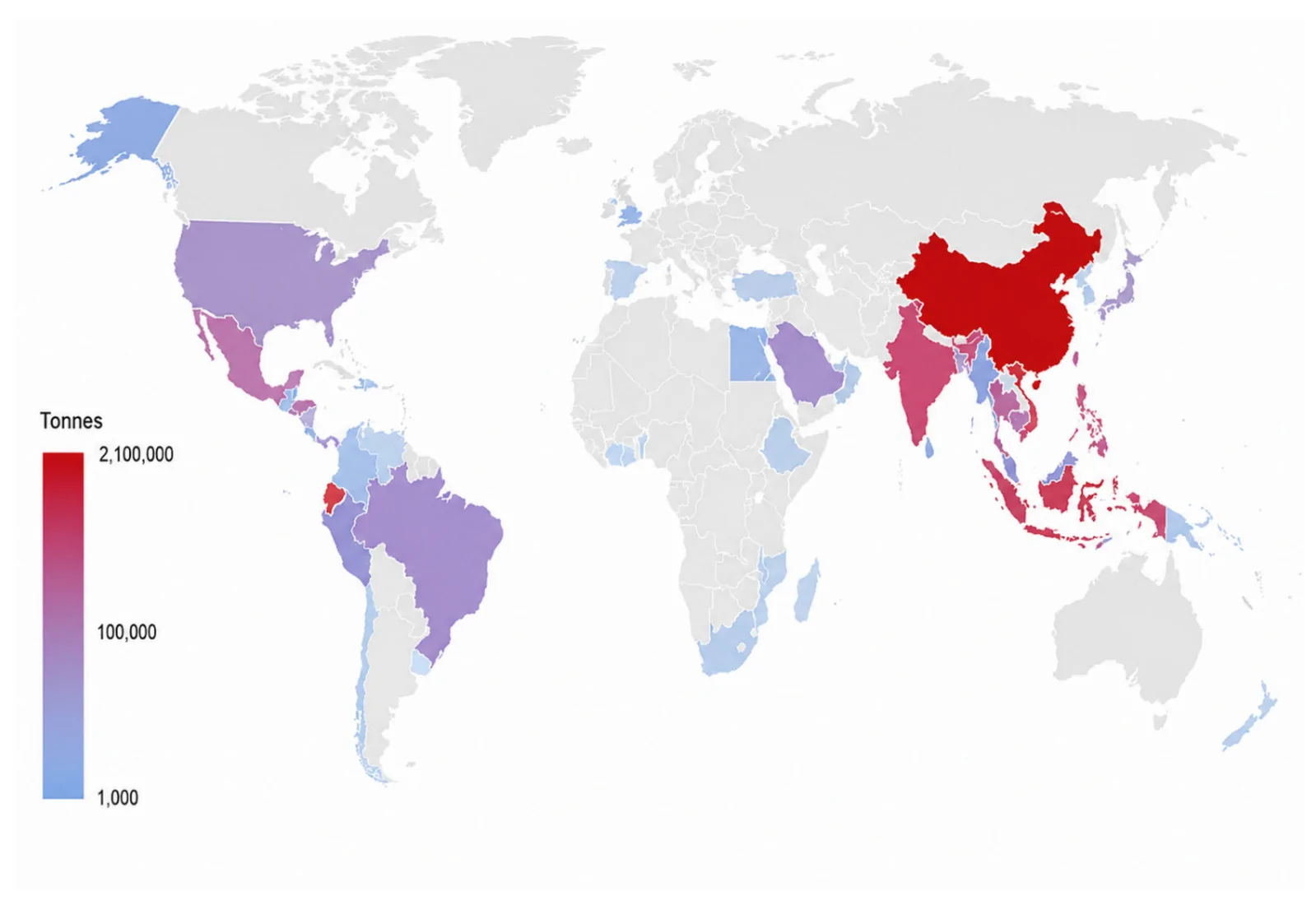
Global distribution of whiteleg shrimp (*Penaeus vannamei*) aquaculture production in 2022. Production data were obtained from the FAO FishStat Global Aquaculture Production database (1950–2022 release) (https://www.fao.org/fishery/fishstat/en) (Accessed 1 September 2026). Gray, unavailable data. Original figure created by the authors with AI-assisted image generation using ChatGPT Plus Images (OpenAI), followed by author review and verification of all scientific content and terminology.

**Figure 3:**
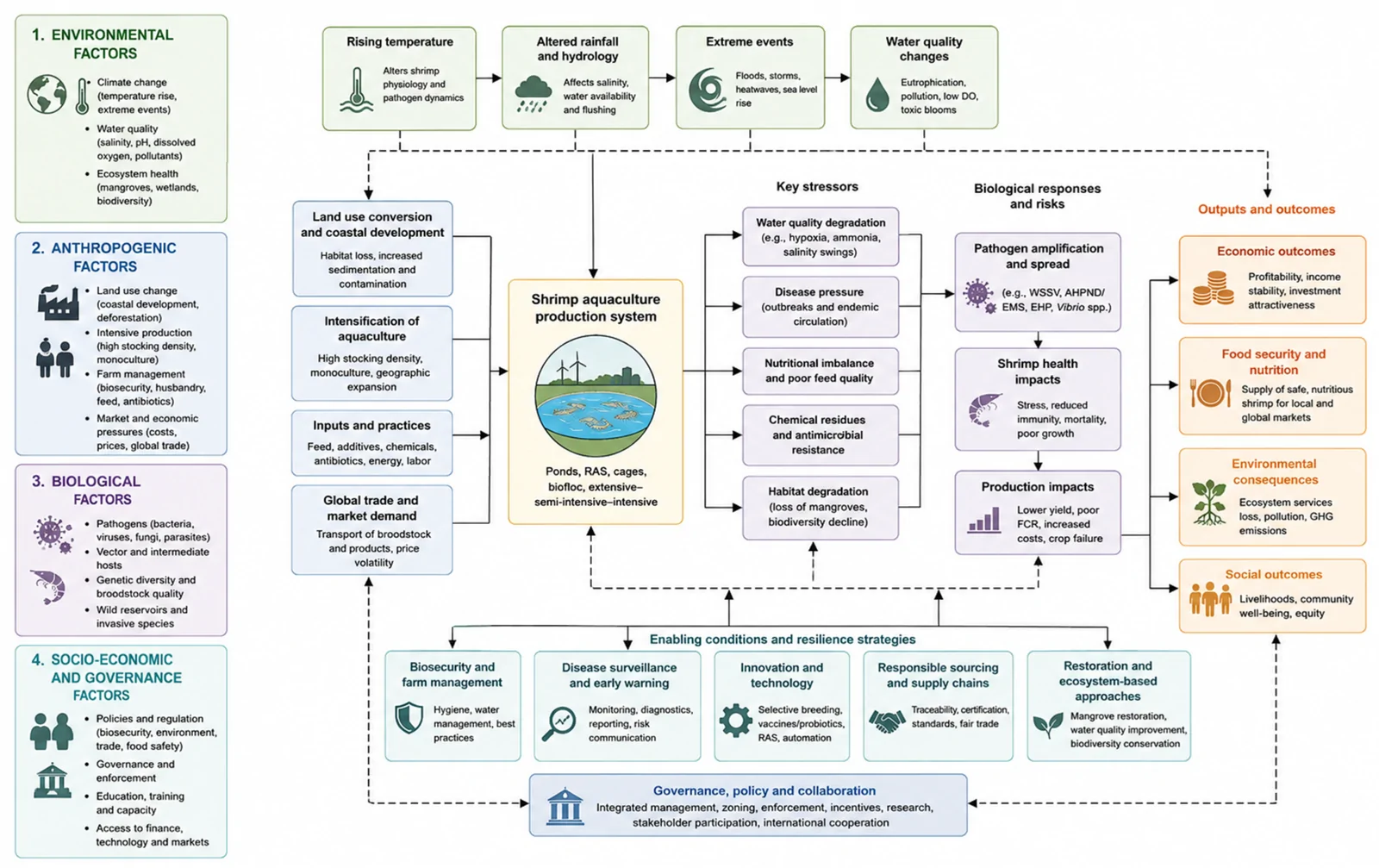
Integrated conceptual framework of drivers, stressors, biological responses, resilience strategies, and outcomes in shrimp aquaculture. Legend: → direct influence, ---> indirect influence/feedback. Abbreviations: AHPND, acute hepatopancreatic necrosis disease; DO, dissolved oxygen; EHP, *Enterocytozoon hepatopenaei*; EMS, early mortality syndrome; FCR, feed conversion ratio; GHG, greenhouse gases; RAS, recirculating aquaculture system; WSSV, white spot syndrome virus. Original figure created by the authors with AI-assisted image generation using ChatGPT Plus Images (OpenAI), followed by author review and verification of all scientific content and terminology.

**Table 1:** One Health dimensions of *Vibrio parahaemolyticus*: interactions across human, animal, and environmental domains.

Domain	Key Drivers/Risk Factors	Examples/Exposure Pathways	Outcomes/Impact	Control and Prevention Strategies
Human health	Consumption of contaminated seafood; immunocompromised status; poor food handling	Raw or undercooked shellfish (oysters, shrimp, fish); wound exposure to seawater	Gastroenteritis, wound infections, sepsis; hospitalization, and mortality	Food safety practices; proper cooking; hygiene; clinical awareness and early diagnosis
Animal health (aquaculture)	Intensive aquaculture practices; high stocking density; water quality fluctuations	Shrimp farming systems; hatcheries; contaminated water sources	Acute hepatopancreatic necrosis disease (AHPND); translucent postlarval disease (TPD); high mortality and economic losses	Biosecurity measures; water quality control; reduced antibiotic use; alternative therapies (e.g., probiotics, bacteriophages)
Environmental health	Climate change; ocean warming; salinity changes; pollution	Coastal and estuarine waters; marine sediments; plankton and shellfish microbiomes	Increased bacterial abundance; expanded geographic distribution; seasonal outbreaks	Environmental monitoring; climate-resilient surveillance systems; water quality management
Food systems	Global seafood trade; supply chain contamination; inadequate storage conditions	Markets, restaurants, street food; cross-contamination during processing	Foodborne outbreaks; public health burden; economic impact on fisheries	Food chain surveillance; cold chain maintenance; regulatory policies; hazard analysis systems (HACCP)
Antimicrobial resistance (cross-cutting)	Overuse of antibiotics in aquaculture and clinical settings	Environmental reservoirs; biofilms; horizontal gene transfer	Emergence of resistant strains; treatment challenges	Antimicrobial stewardship; monitoring resistance patterns; alternative interventions

**Table 2:** Major virulence factors and pathogenic mechanisms of *Vibrio parahaemolyticus*.

Virulence Factor	Gene(s)	Mechanism of Action	Clinical Relevance	Occurrence
Thermostable direct hemolysin (TDH)	tdh	Pore-forming toxin; induces ion flux and chloride secretion in intestinal cells	Major cause of watery diarrhea; key marker of pathogenic strains	Predominantly in clinical isolates (>80%)
TDH-related hemolysin (TRH)	trh	Similar to TDH; associated with urease activity and pH modulation	Contributes to enteropathogenicity and survival in the host	Less frequent; <20% of clinical isolates
Type III secretion system (T3SS1)	Multiple (T3SS1 genes)	Injects effector proteins into host cells causing cytotoxicity	Associated with cell death and tissue damage	Present in most strains
Type III secretion system (T3SS2)	Multiple (T3SS2 genes)	Induces enterotoxicity and intestinal fluid secretion	Strongly linked to gastroenteritis	Mainly in pathogenic strains
Type VI secretion system (T6SS)	Multiple (T6SS genes)	Mediates interbacterial competition and host interaction	May enhance colonization and persistence	Variable distribution
Adhesion factors	Multiple adhesin genes	Promote attachment to host intestinal cells	Essential for colonization and infection establishment	Present in pathogenic strains
Iron acquisition systems	Siderophore-related genes	Facilitates iron uptake under host-limited conditions	Supports bacterial growth and virulence	Widely distributed
Lipopolysaccharides (LPS)	LPS biosynthesis genes	Induces host immune response and inflammation	Contributes to systemic symptoms	Present in all strains
Proteases	Various protease genes	Degrade host proteins and tissues	Facilitates invasion and tissue damage	Variable
Outer membrane proteins (OMPs)	OMP-related genes	Involved in adhesion, immune evasion, and transport	Contribute to virulence and host interaction	Widely distributed
Biofilm formation	Multiple (e.g., EPS-related genes)	Forms protective matrix enhancing survival and resistance	Associated with persistence and antimicrobial resistance	Common in environmental and clinical strains

**Table 3:** Major outbreaks and epidemiological features of *Vibrio parahaemolyticus* worldwide.

Country/Region	Year(s)	Cases/Outbreaks	Source of Infection	Predominant Serotype/Strain	Key Findings
China (Zhejiang)	2010–2022	383 outbreaks; 4382 cases	Seafood, ready-to-eat foods	O3:K6 (predominant)	Strong seasonality (July–September); major cause of bacterial gastroenteritis
China (multiple provinces)	2000–2009	3.11% of 79,075 diarrheal samples	Not specified	O3:K6 and others	Significant pathogen in coastal regions
China (eastern)	2009–2013	563/6951 cases (~8.1%)	Seafood	Not specified	Peak detection in summer (up to 13.1%)
United States	1973–1998	345 cases	Raw oysters	O3:K6 (emerged later)	59% gastroenteritis; high hospitalization (45%)
United States (Pacific NW)	1998	Major outbreak	Oysters	O3:K6	First large outbreak linked to pandemic clone
Chile (Puerto Montt)	2005	>3600 cases	Seafood	O3:K6	One of the largest outbreaks in South America
Chile	2007	475 cases	Seafood	O3:K6 (73%)	Persistence of pandemic clone
Peru	2009	30 isolates	Seafood	O3:K59	Emergence of a new serotype; all TDH+
Mexico	2004–2011	144 clinical strains	Seafood	O3:K6 (66%)	Significant circulation of pandemic clone
Panama	1975	40 cases	Shrimp	Not specified	First reported outbreak in Central America
Germany (Berlin)	2023–2024	56% seafood contamination	Retail seafood	Multiple species	Highlights food safety concerns
South America (general)	Various	Limited data	Seafood, marine environment	Multiple	Underreported but widespread presence
Spain (Galicia)	1999	64 cases	Raw oysters	*tdh*+ pathogenic strains	Major autochthonous European seafood-associated outbreak
Northern Europe (Norway, Denmark, Sweden, Finland, Poland, Estonia)	2018	89 *V. parahaemolyticus* cases	Mainly coastal/marine exposure	Multiple strains	Marked summer increase during exceptional heatwave; 87.6% occurred in summer

**Table 4:** Antimicrobial resistance patterns, mechanisms, and implications in *Vibrio parahaemolyticus*.

Antibiotic/Class	Resistance Pattern	Associated Genes/Mechanisms	Source/Context	Clinical and Public Health Implications
Beta-lactams (e.g., ampicillin)	High resistance	*bla* genes; β-lactamase production	Aquaculture and clinical isolates	Limited effectiveness of first-line therapies
Tetracyclines	Frequent resistance	*tet* genes; efflux pumps	Aquaculture environments	Reduced treatment options; selection pressure in farming
Sulfonamides	Moderate resistance	*sul2* gene	Environmental and aquaculture sources	Persistence of resistant strains in water systems
Chloramphenicol	Variable resistance	*floR* gene	Aquaculture and environmental isolates	Potential resistance spread via mobile elements
Macrolides (e.g., erythromycin)	High resistance	Efflux pumps; ribosomal modification	Clinical and environmental isolates	Limits alternative treatment options
Cephalosporins (e.g., cefazolin)	Resistance reported	β-lactamase-related mechanisms	Environmental isolates	Concern for expanded resistance spectrum
Fluoroquinolones (e.g., ciprofloxacin)	Generally sensitive (emerging resistance reported)	Target mutations; efflux systems	Clinical isolates	Remain the key therapeutic option but require monitoring
Aminoglycosides (e.g., gentamicin)	Generally sensitive	Limited resistance mechanisms reported	Clinical isolates	Effective treatment option
Biofilm-associated resistance	Increased tolerance to multiple antibiotics	Extracellular polymeric substances (EPS); reduced penetration	Environmental and host-associated biofilms	Chronic infection potential; treatment failure
Multidrug resistance (MDR)	Increasing trend	Horizontal gene transfer; plasmids; integrons	Aquaculture and environmental reservoirs	Major One Health concern; complicates control strategies

## Data Availability

No new data were created or analyzed in this study. Data sharing is not applicable to this article.
